# Inclusion of Health in Impact Assessment: A Review of Current Practice in Sub-Saharan Africa

**DOI:** 10.3390/ijerph17114155

**Published:** 2020-06-10

**Authors:** Dominik Dietler, Ruth Lewinski, Sophie Azevedo, Rebecca Engebretsen, Fritz Brugger, Jürg Utzinger, Mirko S. Winkler

**Affiliations:** 1 Swiss Tropical and Public Health Institute, P.O. Box, CH-4002 Basel, Switzerland; ruth.lewinski@gmail.com (R.L.); sazevedo@ethz.ch (S.A.); juerg.utzinger@swisstph.ch (J.U.); mirko.winkler@swisstph.ch (M.S.W.); 2 University of Basel, P.O. Box, CH-4001 Basel, Switzerland; 3 Department of Humanities, Social- and Political Sciences, Swiss Federal Institute of Technology, Clausiusstrasse 37, CH-8092 Zurich, Switzerland; rh.engebretsen@gmail.com (R.E.); fritz.brugger@nadel.ethz.ch (F.B.)

**Keywords:** environmental impact assessment, extractive industry, health impact assessment, low- and middle-income countries, mining sector, sub-Saharan Africa

## Abstract

Natural resource extraction projects, including those in the mining sector, have various effects on human health and wellbeing, with communities in resource-rich areas in sub-Saharan Africa (SSA) being particularly vulnerable. While impact assessments (IA) can predict and mitigate negative effects, it is unclear whether and to what extent health aspects are included in current IA practice in SSA. For collecting IA reports, we contacted 569 mining projects and 35 ministries regulating the mining sector. The reports obtained were complemented by reports identified in prior research. The examination of the final sample of 44 IA reports revealed a heavy focus on environmental health determinants and included health outcomes were often limited to a few aspects, such as HIV, malaria and injuries. The miniscule yield of reports (1.6% of contacted projects) and the low response rate by the contacted mining companies (18%) might indicate a lack of transparency in the IA process of the mining sector in SSA. To address the shortcomings identified, policies regulating IA practice should strengthen the requirements for public disclosure of IA reports and promote a more comprehensive inclusion of health in IA, be it through stand-alone health impact assessment or more rigorous integration of health in other forms of IA.

## 1. Introduction

Impact assessment (IA) is an established approach to minimize adverse environmental, social and health impacts of projects, policies and programs, while fostering opportunities for equitable and sustainable development [1,2,3]. The first legislation promoting IA dates back more than 50 years, when legislation on environmental impact assessment (EIA) was introduced in the United States [3]. Passed in 1969, this legislation required human health to be included as part of the assessment. Since then, the field of IA has evolved and diversified. During the 1970s, the social impact assessment (SIA) approach was established, placing particular emphasis on the interrelations between the environmental and social impacts, including health [1,3]. With the aim to more specifically address potential impacts of projects, programs, plans and policies on human health as a stand-alone process, health impact assessment (HIA) was introduced in the late 1980s/early 1990s [2,4,5,6]. Over the past 30 years, the methodology and approach for assessing health impacts has been further developed [7]. At present, many different forms and typologies of IA exist, including integrated IA, such as environmental and social impact assessments (ESIA), or environmental, social and health impact assessments (ESHIA) [8].

Health aspects lack standardized integration in different forms of IA [3,5]. Broadly speaking, two strategies exist to consider health in the IA process. First, health is considered in a specific HIA, as a comprehensive, stand-alone approach [2]. Second, health can be addressed as part of EIA, or integrated IA, such as environmental and health impact assessment (EHIA) or ESHIA [9,10]. The inclusion of health in EIA holds particular promise, since it is promoted by national legislation in most countries [3]. However, research on the inclusion of health in EIA and other forms of IA from high-income countries shows that health is, in general, insufficiently considered, with the exception of stand-alone HIA [8,11,12,13,14,15,16]. In other forms of IA, the spectrum of health aspects assessed is narrow and centered around environmental determinants of health, often neglecting the various impacts on social and institutional factors that inherently affect health [11,12,13,14,15,16].

It is encouraging that HIA has gained in popularity in the recent past; yet, there are considerable differences regarding the use of HIA from one world region to another [2,17,18]. The practice of HIA is particularly lacking in sub-Sahara Africa (SSA), which might be explained by an absence of legal frameworks promoting HIA and a paucity of trained practitioners [18,19]. The absence of HIA in SSA is an issue, as this region is particularly vulnerable to adverse health impacts governed by social-ecological contexts (e.g., widespread poverty, low capacity of public infrastructure, favorable conditions for the transmission of vector- and water-borne diseases, high prevalence of infectious diseases and vulnerabilities to climate change) [20,21,22]. At the same time, many countries in SSA are rich in natural resources. In turn, there is a large and growing number of projects in the mining sector [23,24]. Their development is associated with a broad range of potential positive and negative impacts on health outcomes (e.g., increased rates of HIV or malaria) [25,26,27,28] and health determinants (e.g., increased household income, increased public education investment, in-migration and environmental degradation) [29,30,31,32,33,34]. The proper management of potential negative impacts of projects in this rapidly growing sector holds promise for improving public health and promoting sustainable development in the mining sector [23,32,35].

Against this background, a thorough assessment of health impacts of mining projects is particularly salient in SSA. However, whether and to what extent and quality health has been included in different forms of IA in the mining sector of SSA needs to be investigated. We analyzed IA reports of mining projects in SSA and determined the scope and quality of the inclusion of health. More specifically, the following research questions were addressed. First, are health aspects included in different types of IA reports and if so, which ones? Second, what kind of data sources are used as evidence-base for HIA or health in other forms of IA?

## 2. Materials and Methods

In this study, IA reports were obtained from several sources. The reports that fulfilled our inclusion criteria described below were systematically screened for specific health aspects.

### 2.1. Strategy for Identification of Relevant Reports

**Figure 1:**
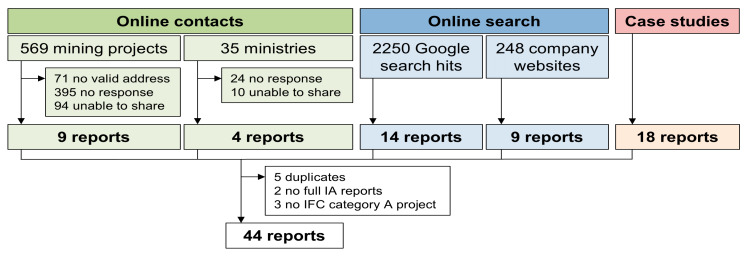
Sources and flow chart of impact assessment reports. IA = impact assessment; IFC = International Finance Corporation

IA reports of large mining projects in SSA were collected from three different sources in order to maximize the number of reports (Figure 1).

#### 2.1.1. Online Contacts within Mining Companies and Ministries

A standardized message (see online Supplementary File S1) was sent to 569 mining projects in SSA that were listed in the Standard & Poor’s (S&P) Global Market Intelligence Mining Database [36] on 26 October 2018. The contacts were asked for access to IA reports, emphasizing strict confidentiality and offering to sign a non-disclosure agreement. Additionally, an adapted version of the message (see online Supplementary File S2) was sent to ministries regulating the mining sector (e.g., Ministry of Mines and Ministry of Environment) in 35 countries of SSA known to host industrial mining projects. For member countries of the Extractive Industries Transparency Initiative (EITI), a request to their contact person was sent. All messages were sent either through a contact form on the company/ministry web page or directly by e-mail. A maximum of two reminders at an interval of at least 2 weeks were sent if the contacts did not respond to the initial message. The messages to the companies were sent between November 2018 and May 2019, those to the ministries and EITI representatives between May and July 2019.

#### 2.1.2. Online Search

Publicly available reports were searched online through Google and company web pages. In the Google search engine, a systematic online search was conducted using Boolean operators. Separately for each country in SSA, the term “impact assessment” and terms representing an activity of natural resource extraction projects (“natural resource OR mine OR mining OR dam OR drilling OR gas OR hydrocarbon OR oil OR petrol OR hydroelectricity OR hydropower OR biofuel OR electricity OR exploration OR exploitation OR extraction”) were combined with the different spellings for the respective country (e.g., “Côte d’Ivoire” OR “Ivory Coast”). Initial piloting of the search methodology revealed that most of the relevant documents were found among the first 50 hits. Of note, this search terminology also served another research component that systematically searched contents of IA reports of a broader spectrum of large natural resource extraction projects [37]. For the current analysis, the full sample of reports retrieved was reduced to include mining projects only. The search was carried out in October and November 2018 in Switzerland. Additionally, the web pages of the contacted companies were visited to check the public availability of IA reports. If no direct link to the company was available in the mining database, the project and the company operating or owning the project were searched on Google. All web pages were visited in May 2019.

#### 2.1.3. Case Studies

An ongoing research initiative, the “health impact assessment for sustainable development” (HIA4SD) project [38,39] aims at generating a deeper understanding of health impacts of natural resource extraction projects in Burkina Faso, Ghana, Mozambique and Tanzania. As part of the research activities, in-country project partners established contacts with mining companies and ministry representatives and obtained reports between March 2018 and January 2019. As a result, IA reports were made available either directly by the companies or by the national environmental authorities.

### 2.2. Screening of IA Reports

In a first step, the eligibility of the reports was assessed. Reports were excluded if (i) not all IA reports were available for projects for which multiple assessments were conducted (e.g., only SIA was available that was conducted in connection with an EIA); (ii) it represented only a summary of the assessment (e.g., environmental impact statement); or (iii) the project was not rated as a category A project according to the International Finance Corporation (IFC)’s environmental and social categorization, so that the sample includes only projects “with potential significant adverse environmental or social risks and/or impacts that are diverse, irreversible, or unprecedented” [40,41]. Category A projects, such as most large-scale mining projects, are required to conduct a comprehensive IA, including a thorough assessment and data collection for informing potential health impacts [40,42]. More specifically, in contexts where availability and quality of health-related data are limited, the collection of primary data in affected communities is indicated for ensuring a robust evidence-base for the IA and enabling monitoring of health impacts over time [43].

The second step comprised of examining the full IA reports for their consideration of different health factors. Additionally, to assess the completeness of the executive summaries, the summaries of the IA reports found through the Google search were screened separately. The screening followed the same methodology for both, the full IA reports and the sample of executive summaries. For each report section (e.g., baseline, impact assessment, mitigation measures and monitoring plan), information on the inclusion of different health aspects was extracted. An adapted analysis framework from Quigley et al. [44], the IFC HIA guidelines [43] and Winkler et al. [20] was used, which comprised 4 health determinant categories (Table A1) and 10 health outcome groups (Table A2). In total, 23 specific health determinants and 35 health outcomes were identified. Furthermore, the data sources that the IAs used for the health baseline assessment were categorized into different primary and secondary data source categories. The primary data sources consisted of key informant interviews (KIIs), focus group discussions (FGDs), household surveys (HHS) and biological or environmental samples, including field observations. The options for the secondary data sources included routine health surveillance data (e.g., health facility data, District Health Information System 2 (DHIS 2) data), national and regional surveys (e.g., Demographic and Health Surveys (DHS) and Multiple Indicator Cluster Surveys (MICS)), official government statistics (national or local), peer-reviewed articles and grey literature. Other data sources that might be relevant were classified as “other primary data source” and “other secondary data source”.

Full reports that were electronically available were screened by two authors (D.D. and R.L.), while executive summaries were examined by a third author (S.A.). Case study reports that were only available in printed form were examined by the HIA4SD project research associates in the respective countries. Parallel screening of the reports and validation of the results ensured the consistent application of the methodology across all assessors. To facilitate data entry during the screening stage, the assessors used an online survey tool (www.surveymonkey.com).

### 2.3. Data Extraction and Analysis

The survey data were extracted and summary statistics generated using R version 3.5.1 (R Foundation for Statistical Computing, Vienna, Austria) [45]. The unit of analysis were the projects. Hence, if more than one IA report was available for a specific project (e.g., a HIA was conducted together with an EIA), the health aspects included in the different reports were combined. The statistics are presented for different aspects for each health determinant and outcome. Comparisons were made between the different report sections and report types (health-specific IA (HIA and ESHIA) vs. non-health-specific IA (EIA, SIA or ESIA)).

## 3. Results

As shown in Figure 1, a total of 54 IA reports were obtained. Reaching out to contacts of 569 mining projects and representatives from ministries in 35 countries of SSA yielded only 9 and 4 reports, respectively. Through the systematic Google search, 14 reports were found. Additionally, the IA reports of 9 companies were readily available on company web pages. The sample was completed by 18 reports obtained from case studies in the HIA4SD project. Among the case study reports, 2 were also found on the company web pages and 2 were made available by company contacts. Furthermore, 1 report was shared directly by a company contact and publicly on the web page. Two reports were excluded from the analysis because only part of the IA documents were available. Additionally, 3 reports considered only the expansion of existing projects and, thus, did not necessarily require a full IA (i.e., not category A projects). Our final sample included 44 IA reports.

### 3.1. Report Characteristics

**Figure 2:**
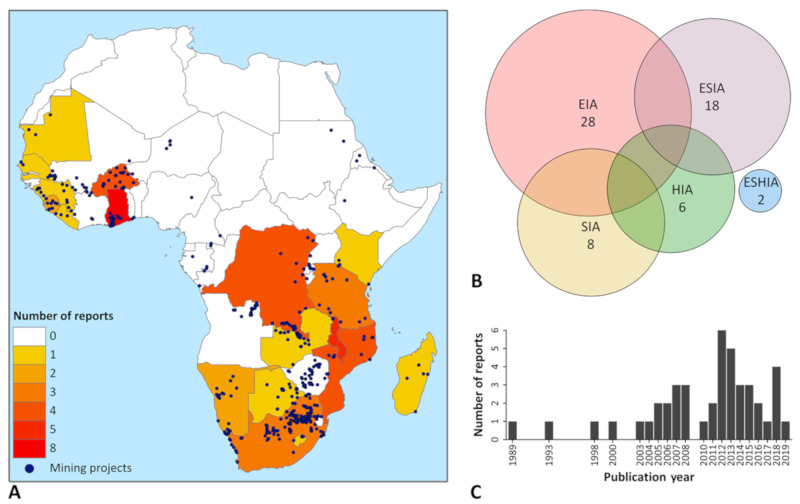
Characteristics of the 44 included impact assessment (IA) reports. (**A**) Country of included reports and location of contacted projects, listed in the Standard & Poor’s Global market intelligence mining database [36] on 26 October 2018; (**B**) type of report (overlaps indicate projects for which more than one type of IA was conducted); (**C**) publication year. EIA = environmental impact assessment; ESHIA = environmental, social and health impact assessment; ESIA = environmental and social impact assessment; HIA = health impact assessment; SIA = social impact assessment

Panel A in Figure 2 shows the geographic distribution of the 44 included IA reports as well as the location of the 569 contacted mining projects in SSA. Reports from 18 different countries were obtained. Most reports stemmed from the HIA4SD project countries, namely Ghana (n = 8), Burkina Faso (n = 4), Mozambique (n = 4) and Tanzania (n = 3). Furthermore, a sizable number of reports of projects in Malawi (n = 5) and the Democratic Republic of the Congo (n = 4) were shared. Of note, despite hosting the vast majority of mines listed in the S&P mining database (n = 263) very few reports (n = 3) could be retrieved from South Africa.

A broad variety of IA report types were collected (see Figure 2, Panel B). For some projects, more than one type of IA report was available. Most of the reports were EIAs (n = 28), which were often conducted alongside SIA, HIA and ESIA. Only 8 reports were obtained that addressed health by design (i.e., HIA and ESHIA).

A temporal pattern is visible in the publication year of the IA reports (see Figure 2, Panel C). Most of the reports were published in 2010 or later (n = 28). Only 3 of the reports were published before 2000.

### 3.2. Inclusion of Health Aspects

#### 3.2.1. Inclusion of Health Determinants

**Figure 3:**
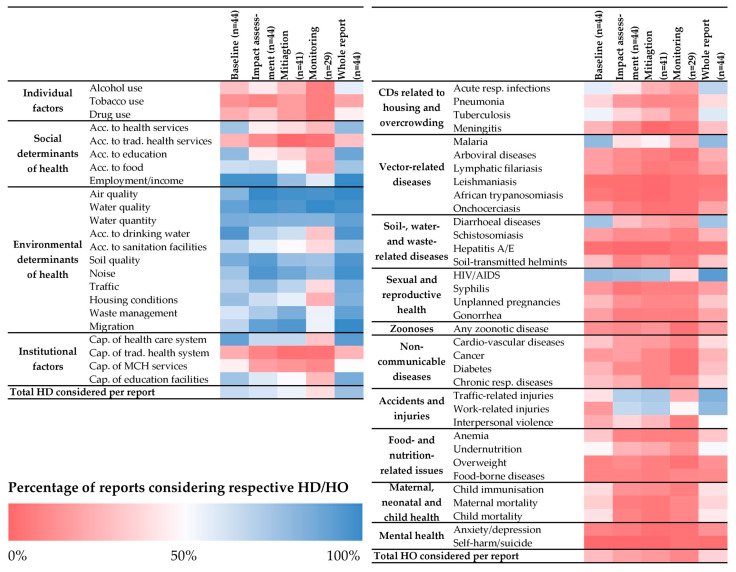
Inclusion of health determinants (HD; left panel) and health outcomes (HO; right panel) in impact assessment reports. Colors represent the percentage of reports or report sections considering the specific health aspect. Red shading indicates percentages below 50%, blue shadings above 50%. Acc. = access; Cap. = capacity; CD = communicable disease; MCH = maternal and child health; resp. = respiratory; trad. = traditional.

Figure 3 provides an overview of the percentage of IA reports considering the screened health determinants. Large differences were observed between the health determinants. While the environmental determinants were considered in most IA reports, the social determinants and institutional factors were less often included. Some particular aspects received little attention, including the capacity of maternal and child health services, as well as access and capacity of traditional health services. The impacts on individual health risk factors, such as alcohol consumption, tobacco or drug use, were least frequently assessed.

Overall, the number of health determinants considered decreased with later sections of the IA reports (i.e., mitigation and monitoring plan). The average percentages of health determinant items included were 65.4%, 61.2%, 54.7% and 39.3% in the baseline description, impact assessment section, mitigation plan and monitoring plan, respectively (see Table A3).

#### 3.2.2. Inclusion of Health Outcomes

Health outcomes were less frequently included in the IA reports than health determinants (Figure 3 and Table A4). Overall, a third (35.9%) of health outcomes were considered across the report sections, compared to 76.8% for the health determinants. In the IA chapters, only 19.4% of health outcomes were included.

Only 8 health outcomes were included in more than 50% of the reports. Among them were, in decreasing order, HIV/AIDS, traffic-related injuries, work-related injuries, malaria, diarrhea, acute respiratory infections, tuberculosis and undernutrition. Zoonoses, mental health, non-communicable diseases and vector-borne diseases other than malaria received less attention.

Similarly to the health determinants, health outcomes were more often considered in the baseline and impact assessment chapters than in the mitigation and monitoring plans. Mitigation measures for specific health outcomes were proposed in few of the IA reports.

### 3.3. Data Sources

**Figure 4:**
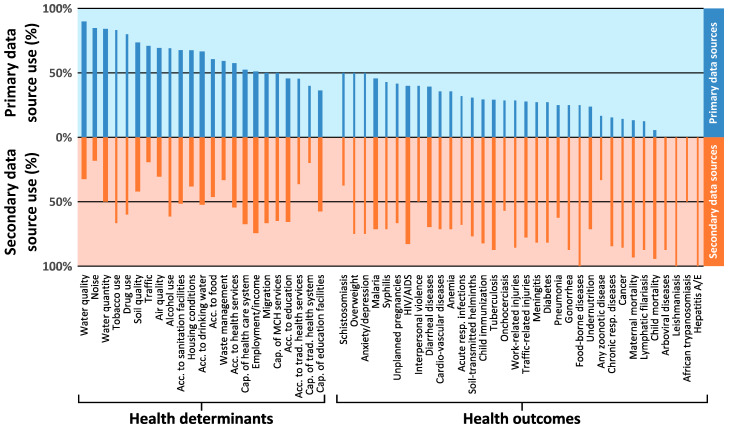
Data sources used for assessing health aspects in impact assessment reports. The height of the bars indicate the percentage of reports using any primary (blue bars) and any secondary (red bars) data source for the different health aspects. Bar widths indicate the number of reports considering the specific health aspect (used as denominator for determining the bar height of the respective aspect). Acc. = access; Cap. = capacity; MCH = maternal and child health; resp. = respiratory; trad. = traditional

Figure 4 shows the percentages of different data sources used as baseline indicators among the IA reports considering the respective health determinants or outcomes. Overall, primary data were collected predominantly for the health determinants. For measuring health outcome indicators, primarily secondary data sources were used. Collection of primary data pertaining on baseline conditions among the potentially affected communities through participatory approaches, such as KIIs, FGDs or HHS, was rare (see also Table A5). For all health-related aspects, peer-reviewed literature was consulted in only a few instances.

For the assessment of environmental determinants (e.g., air quality, water quality and quantity or noise) a comprehensive sample collection was often conducted. In some cases, these aspects were even assessed in separate specialist reports. In contrast, qualitative information from KIIs and FGDs were more often used to assess the social determinants of health. For some aspects related to access and capacity of public services (e.g., health and education), secondary data, such as official statistics, were also used.

In most cases, secondary information for the baseline of specific health outcome indicators stemmed from health facility data or official statistics. If primary data were used, it was mostly qualitative data obtained from KIIs or FGDs.

### 3.4. Comparison between IA Report Types

**Figure 5:**
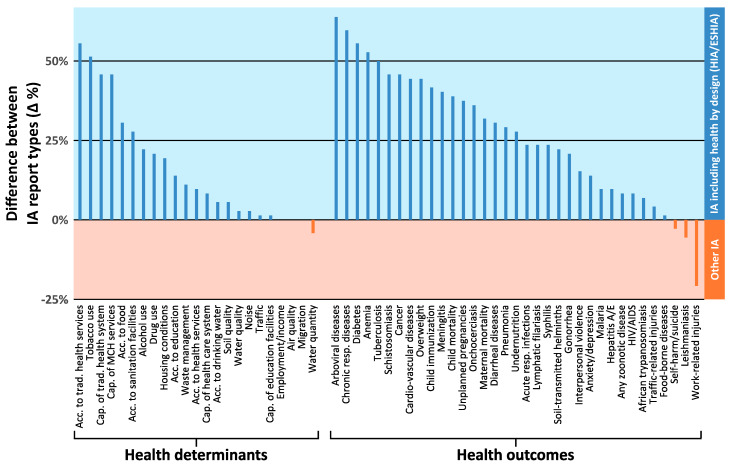
Difference in percentages of impact assessment (IA) reports including the different health determinants and health outcomes between health-specific IA reports and non-health-specific IA reports. Blue bars indicate more frequent consideration of the respective health determinant/health outcome in health-specific IA reports; red bars indicate more frequent consideration in non-health-specific IA reports. Missing bars indicate a difference of 0%. Acc. = access; Cap. = capacity; ESHIA = environmental, social and health impact assessment; HIA = health impact assessment; MCH = maternal and child health; resp. = respiratory; trad. = traditional.

The differences in the percentages of IA reports addressing the various health aspects in health-specific IA (i.e., HIA and ESHIA; n = 8) and non-specific IA (i.e., EIA, ESIA and SIA; n = 36) are shown in Figure 5. Almost all health determinants and outcomes were more prominently featured in the IA reports addressing health by design. Among the health determinants, aspects related to access and capacity of traditional health services were included more frequently in health-specific IA reports. The differences were less pronounced for the environmental determinants of health. With regards to the health outcomes, 32 of 35 studied items were more often considered in projects for which a health-specific IA was conducted. Differences of at least 50 percentage points were observed for tuberculosis, arboviral diseases (e.g., chikungunya, dengue and yellow fever), the non-communicable diseases diabetes and chronic respiratory diseases, anemia and tuberculosis. On the other hand, work-related injuries were featured more often in projects for which no health-specific IA was conducted.

### 3.5. Completeness of Executive Summaries

**Figure 6:**
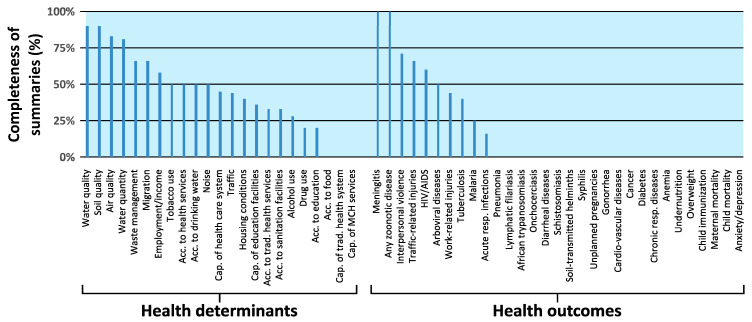
Inclusion of health aspects among the 12 analyzed executive summaries of IA reports. Percentages (bar heights) indicate the number of executive summaries addressing the health aspects relative to the number of full texts considering that aspect. Bar widths indicate the number of full texts addressing the respective health aspect (used as denominator for the bar heights). Missing bars indicate 0% inclusion in the summaries. Acc. = access; Cap. = capacity; MCH = maternal and child health; resp. = respiratory; trad. = traditional

The representation of health aspects in the executive summaries of the IA reports was analyzed and compared to their corresponding full reports (Figure 6). The executive summaries frequently omitted information on the different health determinants and health outcomes, although they were included in the full texts. Similar to the full texts, the executive summaries mainly featured information on environmental determinants of health. Some health outcome categories, such as soil-, water- and waste-related diseases, non-communicable diseases, food- and nutrition-related diseases, maternal and child health or mental health, were not included in the executive summaries despite some full reports having considered these aspects (indicated as missing bars in Figure 6). Leishmaniasis, hepatitis A/E, food-borne diseases and self-harm/suicide were excluded from this analysis because they were not considered in any of the full texts.

## 4. Discussion

Overall, 44 IA reports from 18 countries in SSA were obtained from various sources and analyzed for the inclusion of health. We reached out to as many as 569 mining projects and 35 ministries. However, only 13 reports were obtained from these contacts and sources. Public access to IA reports on the internet was also limited; only 21 IA reports were readily accessible online. Screening of the reports revealed a heavy focus on environmental determinants of health. Health outcomes were considered to a lesser extent than the health determinants. Still, some health outcomes, such as malaria, HIV, diarrheal diseases or injuries, were more frequently included. Furthermore, other health aspects, such as zoonoses, mental health issues, non-communicable diseases and food- and nutrition-related issues, received little attention. Reports that had a specific focus on health (i.e., HIA and ESHIA) addressed substantially more health aspects than other reports. Primary data were frequently collected along with secondary data as indicators for the health determinants, particularly for environmental factors. For health outcomes, primary data collection was the exception rather than the norm. Participatory data collection approaches with affected communities through KIIs, FGDs or HHS were rarely conducted.

The IFC’s Sustainability Framework through its Performance Standards on Environmental and Social Sustainability sets out the requirements for the management of environmental and social risks of industrial investment projects [42]. The IFC Performance Standards have been adopted by the Equator Principles Financial Institutions (EPFI), a consortium that currently embraces more than 100 banks and financial institutions [46,47]. Since the IFC’s Sustainability Framework is considered an international benchmark for identifying and managing environmental, social and health risks [48,49], this standard is also applied in this discussion chapter for reflecting on our findings stemming from a comprehensive review of the available IA reports.

### 4.1. Lack of Transparency

The IFC Performance Standards require projects to publicly disclose information on project-related risks and impacts to affected communities [42]. The scope of this information can range from full IA reports to short summaries of findings, depending on the project size and magnitude of anticipated impacts [42]. For IFC-funded projects, the bank itself publishes a summary of the main findings of the IA [50]. In our study, only a miniscule 1.6% of the 569 contacted large-scale mining projects shared their report, while more than 80% did not respond at all to our data inquest, despite an offer of strict confidentiality. The extremely low yield of IA reports indicates that there is a lack of transparency in current IA practice in the mining sector of SSA.

Research on public disclosure in IA practice from low-human development index (HDI) countries is scarce. In Myanmar, a lack of public disclosure of EIA reports conducted for the oil and gas sector was described, although improvement has been seen in recent years [51]. Instead of disclosing the full IA reports, often, only the executive summaries are published, thereby fulfilling the minimum requirements set out in the IFC Performance Standards. However, our results indicate that these summaries do not offer sufficient insights to inform the public about the potentially broad set of impacts on health. Hence, more stringent requirements for public disclosure of the full IA reports would contribute to increase the accountability of large industrial mining companies and other large-scale infrastructure projects [51]. Hence, in addition to legal texts regulating IA practice, the need for public disclosure of full IA reports for projects should also be more explicitly demanded in policies and guidelines of international financing institutions (e.g., IFC), industry peak bodies (e.g., International Council on Mining and Metals) and private companies.

### 4.2. Narrow Range of Health Aspects Considered

For large-scale projects (i.e., category A) the IFC Performance Standards [42] and the World Bank’s operational policies [40] further require a comprehensive assessment of the project impacts, including aspects of human health and safety. Furthermore, different guidance and scientific documents promote a comprehensive approach to health in HIA, covering the full spectrum of aspects determining human health, especially in complex social-ecological contexts of SSA [44,52,53]. In our sample of IA reports, on average only about a third of investigated health outcomes were included and among the health determinants there was a strong focus on the physical environment. Moreover, when health was integrated in other types or IAs (i.e., EIA, ESIA and SIA), a more narrow range of health aspects were covered. This pattern has been seen in other parts of the world. For example, a lack of inclusion of health aspects was found in EIA reports from the United States [11], Australia [12,15,16] and Vietnam [54]. Furthermore, the assessment of health impacts within EIAs from Australia was mainly limited to risks related to the physical environment [12]. Consistently, in HIAs from low- and middle-income countries, a lack of consideration of the social determinants of health was seen [55]. This may be linked to the limited technical expertise to conduct HIA in many parts of the world [7]. In order to address this constraint, HIA capacity building efforts are needed that do not only aim to build up technical capacity among IA practitioners but also provide trainings to regulators in governments and international financing institutions to appraise IA reports from a health perspective [7,53]. The strengthening of regulatory frameworks that specify under what circumstances HIA is required, and to what extent, could be an important initial step for triggering the demand in HIA capacity building in resource-rich countries of SSA [7,18]. Finally, in light of the health aspects currently not included in IA practice, it should be reflected whether national and international IA guidance documents provide sufficient details on the scope of health to be considered in the IA process.

### 4.3. Lack of Primary Data Collection

A comprehensive assessment of health impacts, as required by the IFC Performance Standards, comprises data collection on health aspects in affected communities [42,43]. Particularly in mining areas in low-HDI countries, the demographic, social-economic, environmental and epidemiological characteristics further warrant the collection of additional local-level data [56]. However, in the IA reports obtained and scrutinized in the present study, primary data were predominantly collected for aspects related to the physical environment. For health outcomes, the assessments often relied on secondary data sources, such as coarse national and regional-level statistics or local health facility data. Although these data sources hold considerable potential for monitoring health indicators, they are prone to low data quality [55,57]. The collection of local-level data by means of KIIs, FGDs and HHS is an additional means to engage affected groups in the IA process and can help identify and address local health impacts among vulnerable and marginalized populations [58,59,60,61,62,63]. Comprehensive baseline health data collection requires broad public health expertise among practitioners conducting the IA [26,64,65]. However, health specialists in countries of SSA are rarely engaged in IA and often have limited awareness and knowledge about the IA process [19]. For the health sector to be more actively engaged in HIA, capacity building efforts should reach out beyond the public health sector (e.g., actors in overseeing ministries) to increase the understanding of the skill set required for conducting a thorough assessment of health impacts [19,53].

### 4.4. Limitations

For this study, we attempted to pursue the different options that affected community members have at their disposal for accessing IA reports. Physical contacts with project proponents or local authorities within the countries may potentially have increased the yield of reports. However, given that only 18% of companies responded to our data inquiry indicates that project representatives are difficult to approach. The resulting small and geographically clustered sample of IA reports limits the representativeness of our sample from which we derive our conclusions.

Furthermore, the analysis only assessed whether and to what extent health issues were addressed. An analysis of the interrelationships between the different health aspects or of the quality of the assessment itself (e.g., the necessity of primary data collection) was beyond the scope of our study. For conclusively judging the appropriate use of different data sources, a more in-depth study is needed, taking into account local characteristics and the quality of alternative data sources.

## 5. Conclusions

This comprehensive review of IA reports of mining projects in SSA points at three main shortfalls of current IA practice: (i) lack of transparency; (ii) narrow scope of considered health aspects, with a strong focus on the physical environment; and (iii) lack of local-level primary data collection on health outcomes. There are different potential approaches to address these shortcomings at the national and international level. At the national level, ministries overseeing IA should reconsider how health is addressed in regulatory frameworks and policies regulating IA practice. This should include critical reflections on whether there is sufficient specificity provided in terms of methodological guidance on how to assess health impacts (i.e., the width (range of potential impacts) and depth (quality of the evidence-base) of the assessment) either in HIA as a stand-alone approach or integrated in other forms of IA. Furthermore, there is a need to understand whether existing frameworks provide sufficient guidance as to which expertise is needed for leading the assessment of health impacts. In addition, regulatory frameworks should be revised if they do not sufficiently promote disclosure of IA findings, with particular considerations for health-related information.

At the international level, financing institutions, such as the IFC and the members of the EPFI, can play a crucial role in closing the identified gaps. This can be done by setting and enforcing more stringent requirements for public disclosure of full IA reports along with strengthening guidance on how health needs to be included in different forms of IA in order to achieve consistency in quality. Finally, any efforts in promoting more rigorous inclusion of health in IA must be coupled with HIA capacity building, which appears particularly salient in the currently environment-dominated impact assessment practice in SSA. Improving international standards for HIA lays a foundation to improve global relationships; health outcomes for local communities need to be prioritized in order to create long-term, sustainable economic investment opportunities. We encourage other groups who pursue IA in the mining and other sectors in SSA and elsewhere to specifically address health, which cannot be emphasized enough in the current COVID-19 pandemic.

## Supplementary Materials

Supplementary materials can be accessed at: https://www.mdpi.com/1660-4601/17/11/4155/s1.

## Appendix A

**Table A1:** Health determinant categories.

Health Determinant Categories	Description
Individual factors	Factors related to the individual’s biology and behavior. These comprise for example gender, age, ethnicity, dietary intake, level of physical activity, tobacco use, alcohol intake, personal safety, sense of control over own life, employment status, educational attainment, self-esteem, life skills, stress levels, resilience and risk behavior.
Social determinants of health	Conditions in which people are born, grow, live, work and age. These include access to services and community (health, education, nutrition, institutional and social support, social and health insurance); income/unemployment rate; distribution of wealth; empowerment of women; sexual customs and tolerance; racism; attitudes to disability; trust; sites of cultural and spiritual significance.
Environmental determinants of health	Physical, chemical, and biological factors external to a person, and all the related factors impacting behaviors, such as exposure to heavy metals, pesticides and other compounds, solvents or spills and releases from road traffic; air pollution (indoor and outdoor); noise pollution and exposure to malodors. It also includes factors, such as inadequate housing, water and sanitation services, and the mixing of population groups with different levels of communicable diseases which can be associated with in-migration.
Institutional factors	Availability of services, including (traditional) health services, transport and communication networks; educational and employment; environmental and public health legislation; environmental and health monitoring systems; laboratory facilities; social and health insurance schemes.

**Table A2:** Health outcome categories.

Health Outcome Categories	Description
Communicable diseases related to housing and overcrowding	Transmission of communicable diseases (e.g., acute respiratory infections, pneumonia, tuberculosis, meningitis, plague, leprosy, etc.) that can be linked to inadequate housing design, overcrowding and housing inflation
Vector-related diseases	Mosquito, fly, tick and lice-related diseases (e.g., malaria, dengue, yellow fever, lymphatic filariasis, leishmaniasis, human African trypanosomiasis, onchocerciasis, etc.)
Soil-, water- and waste-related diseases	Diseases that are transmitted directly or indirectly through contaminated water, soil or non-hazardous waste (e.g., diarrheal diseases, schistosomiasis, hepatitis A and E, poliomyelitis, soil-transmitted helminthiases, etc.)
Sexual and reproductive health	Sexually-transmitted infections such as syphilis, gonorrhea, Chlamydia, hepatitis B and, most importantly, HIV/AIDS
Veterinary medicine and zoonotic diseases	Diseases affecting animals (e.g., bovine tuberculosis, swinepox, avian influenza) or that can be transmitted from animal to human (e.g., rabies, brucellosis, Rift Valley fever, monkey pox, Ebola, leptospirosis, etc.)
Non-communicable diseases	Cardiovascular diseases, cancer, diabetes, that can be linked to changes in lifestyle, exposure to hazardous materials in air, water or soil, and noise
Accidents/injuries	Road traffic or work-related accidents and injuries (home and project related); drowning; unintentional poisoning
Food- and nutrition-related issues	Adverse health effects such as malnutrition, anemia, micronutrient deficiencies or obesity due to e.g., changes in agricultural and subsistence practices, or food inflation; gastroenteritis, food-borne trematodiases, etc.
Maternal and child health	Prenatal, natal and postpartum health conditions, infant and child health and immunization
Mental health	Psychological health conditions linked to resettlement of populations or changes in lifestyles (e.g., anxiety, depression, stress symptoms, suicide)

**Table A3:** Inclusion of health determinants in impact assessment reports.

Health Determinant Categories		Baseline (n = 44)	Impact Assessment (n = 44)	Mitigation (n = 41)	Monitoring (n = 29)	Whole Report (n = 44)
**Individual factors**	Alcohol use	29.5	43.2	26.8	6.9	56.8
	Tobacco use	13.6	9.1	17.1	6.9	20.5
	Drug use	22.7	31.8	17.1	6.9	45.5
**Social determinants of health**	Access to health services	75.0	45.5	39.0	27.6	79.5
	Access to trad. health services	25.0	11.4	0	3.4	29.5
	Access to education	79.5	45.5	36.6	24.1	88.6
	Access to food	63.6	65.9	51.2	20.7	75.0
	Employment/income	97.7	97.7	78.0	58.6	100
**Environmental determinants of health**	Air quality	81.8	100	97.6	96.6	100
	Water quality	90.9	98.0	95.1	100	98.0
	Water quantity	86.4	84.1	82.9	82.8	90.9
	Access to drinking water	95.5	70.5	63.4	31.0	95.5
	Access to sanitation facilities	70.5	56.8	48.8	37.9	77.3
	Soil quality	86.4	93.2	78.0	75.9	95.5
	Noise	75.0	95.5	87.8	79.3	97.7
	Traffic	70.5	79.5	68.3	37.9	86.4
	Housing conditions	77.3	63.6	56.1	24.1	84.1
	Waste management	61.4	72.7	85.4	55.2	90.9
	Migration	68.2	90.9	95.1	55.2	100
**Institutional factors**	Cap. of health care system	90.9	65.9	65.9	31.0	93.2
	Cap. of traditional health system	22.7	9.1	2.4	3.4	25.0
	Cap. of MCH services	45.5	18.2	14.6	10.3	50.0
	Cap. of education facilities	75.0	59.1	51.2	27.6	86.4
**Total health determinants considered per report**		65.4	61.2	54.7	39.3	76.8

Cap. = capacity; MCH = maternal and child health; trad. = traditional.

**Table A4:** Inclusion of health outcomes in impact assessment reports.

Health Outcome Categories		Baseline (n = 44)	Impact Assessment (n = 44)	Mitigation (n = 41)	Monitoring (n = 29)	Whole Report (n = 44)
**Communicable diseases related to housing and overcrowding**	Acute respiratory infections	56.8	43.2	24.4	17.2	68.2
	Pneumonia	36.4	15.9	9.8	10.3	38.6
	Tuberculosis	54.5	36.4	26.8	13.8	59.1
	Meningitis	25.0	11.4	0	3.4	29.5
**Vector-related diseases**	Malaria	79.5	40.9	46.3	24.1	79.5
	Arboviral diseases	18.2	11.4	7.3	3.4	22.7
	Lymphatic filariasis	18.2	11.4	4.9	6.9	18.2
	Leishmaniasis	2.3	2.3	0	3.4	4.5
	African trypanosomiasis	4.5	2.3	0	3.4	6.8
	Onchocerciasis	15.9	6.8	2.4	3.4	20.5
**Soil-, water- and waste-related diseases**	Diarrheal diseases	75.0	29.5	22.0	17.2	75.0
	Schistosomiasis	18.2	11.4	12.2	6.9	25.0
	Hepatitis A/E	2.3	0	0	3.4	4.5
	Soil-transmitted helminths	29.5	9.1	14.6	6.9	31.8
**Sexual and reproductive health**	HIV/AIDS	79.5	77.3	75.6	37.9	93.2
	Syphilis	15.9	4.5	7.3	6.9	18.2
	Unplanned pregnancies	27.3	13.6	9.8	10.3	31.8
	Gonorrhea	18.2	6.8	7.3	6.9	20.5
**Zoonoses**	Any zoonotic disease	13.6	11.4	14.6	3.4	18.2
**Non-communicable diseases**	Cardio-vascular diseases	31.8	18.2	17.1	10.3	38.6
	Cancer	15.9	18.2	9.8	3.4	25.0
	Diabetes	25.0	11.4	9.8	3.4	29.5
	Chronic respiratory diseases	29.5	22.7	9.8	13.8	38.6
**Accidents and injuries**	Traffic-related injuries	40.9	70.5	73.2	24.1	84.1
	Work-related injuries	15.9	65.9	70.7	48.3	79.5
	Interpersonal violence	22.7	36.4	26.8	6.9	50.0
**Food- and nutrition-related issues**	Anemia	31.8	9.1	7.3	6.9	31.8
	Undernutrition	47.7	25.0	22.0	13.8	52.3
	Overweight	9.1	11.4	7.3	3.4	13.6
	Food-borne diseases	9.1	9.1	7.3	10.3	11.4
**Maternal, neonatal and child health**	Child immunization	38.6	13.6	12.2	6.9	40.9
	Maternal mortality	34.1	6.8	4.9	10.3	36.4
	Child mortality	40.9	9.1	4.9	10.3	43.2
**Mental health**	Anxiety/depression	9.1	6.8	2.4	3.4	13.6
	Self-harm/suicide	0	0	0	3.4	2.3
**Total health outcomes considered per report**		28.4	19.4	16.3	10.5	35.9

**Table A5:** Data sources for health determinants and health outcomes used for measuring baseline conditions in impact assessment reports.

Health Determinant and Health Outcome Categories		Any Primary Data Source	Any Secondary Data Source	Key Informant Interviews	Focus Group Discussions	Household Surveys	Env. Sample, Observation	Routine Health Surveillance	National/Re-gional Surveys	Official Statistics	Peer-Reviewed Literature	Grey Literature	Unknown, Other
**Individual factors**	Alcohol use (*n* = 13)	69.2	61.5	38.5	38.5	30.8	7.7	15.4	7.7	23.1	0	23.1	0
	Tobacco use (*n* = 6)	83.3	66.7	33.3	16.7	50	16.7	16.7	33.3	33.3	0	16.7	0
	Drug use (*n* = 10)	80	60	50	40	30	10	20	0	30	0	20	0
**Social determinants of health**	Access to health services (*n* = 33)	57.6	54.5	24.2	24.2	21.2	6.1	18.2	12.1	24.2	3	15.2	24.2
	Access to trad. health services (*n* = 11)	45.5	36.4	27.3	9.1	27.3	9.1	9.1	0	18.2	9.1	9.1	27.3
	Access to education (*n* = 35)	45.7	65.7	17.1	11.4	25.7	5.7	2.9	5.7	42.9	2.9	28.6	25.7
	Access to food (*n* = 28)	60.7	46.4	25	25	28.6	17.9	0	7.1	21.4	0	25	17.9
	Employment/income (*n* = 43)	51.2	74.4	23.3	18.6	27.9	4.7	2.3	9.3	44.2	0	34.9	27.9
**Environmental determinants of health**	Air quality (*n* = 36)	69.4	30.6	5.6	8.3	11.1	58.3	0	0	2.8	0	27.8	22.2
	Water quality (*n* = 40)	90	32.5	10	12.5	7.5	82.5	0	2.5	5	2.5	27.5	10
	Water quantity (*n* = 38)	84.2	50	10.5	10.5	10.5	63.2	0	5.3	13.2	2.6	36.8	10.5
	Access to drinking water (*n* = 42)	66.7	52.4	26.2	14.3	28.6	28.6	0	9.5	23.8	0	33.3	14.3
	Access to sanitation facilities (*n* = 31)	67.7	51.6	25.8	22.6	32.3	16.1	3.2	12.9	29	0	29	9.7
	Soil quality (*n* = 38)	73.7	42.1	13.2	10.5	2.6	65.8	0	5.3	7.9	5.3	28.9	10.5
	Noise (*n* = 33)	84.8	18.2	9.1	9.1	15.2	75.8	0	3	3	0	15.2	12.1
	Traffic (*n* = 31)	71	19.4	19.4	6.5	6.5	45.2	0	3.2	6.5	0	12.9	19.4
	Housing conditions (*n* = 34)	67.6	38.2	20.6	14.7	26.5	20.6	0	11.8	11.8	0	26.5	17.6
	Waste management (*n* = 27)	59.3	33.3	25.9	14.8	18.5	3.7	0	3.7	14.8	0	18.5	22.2
	Migration (*n* = 30)	50	66.7	23.3	6.7	20	3.3	0	10	36.7	3.3	33.3	16.7
**Institutional factors**	Capacity of health care system (*n* = 40)	52.5	67.5	20	15	7.5	22.5	15	5	25	0	30	22.5
	Capacity of trad. health system (*n* = 10)	40	20	20	20	0	0	0	0	10	0	10	50
	Capacity of MCH services (*n* = 20)	50	65	25	5	10	20	15	5	40	5	15	5
	Capacity of education facilities (*n* = 33)	36.4	57.6	18.2	9.1	0	18.2	3	0	33.3	0	27.3	21.2
**CDs related to housing and overcrowding**	Acute respiratory infections (*n* = 3)	32	68	16	16	12	0	36	4	20	4	20	32
	Pneumonia (*n* = 16)	25	62.5	6.2	6.2	12.5	0	37.5	6.2	25	6.2	6.2	31.2
	Tuberculosis (*n* = 24)	29.2	87.5	12.5	8.3	12.5	0	33.3	8.3	25	4.2	33.3	20.8
	Meningitis (*n* = 11)	27.3	81.8	18.2	9.1	9.1	0	18.2	9.1	27.3	0	27.3	0
**Vector-related diseases**	Malaria (*n* = 35)	45.7	71.4	17.1	25.7	25.7	5.7	31.4	5.7	22.9	2.9	28.6	34.3
	Arboviral diseases (*n* = 8)	0	87.5	0	0	0	0	37.5	0	25	12.5	12.5	12.5
	Lymphatic filariasis (*n* = 8)	12.5	87.5	0	0	12.5	12.5	50	0	50	25	12.5	12.5
	Leishmaniasis (*n* = 1)	0	100	0	0	0	0	0	0	100	0	0	0
	African trypanosomiasis (*n* = 2)	0	50	0	0	0	0	0	0	0	0	50	50
	Onchocerciasis (*n* = 7)	28.6	57.1	14.3	0	14.3	0	28.6	0	28.6	0	14.3	14.3
**Soil-, water- and waste-related diseases**	Diarrheal diseases (*n* = 33)	39.4	69.7	12.1	15.2	24.2	0	33.3	6.1	15.2	0	27.3	24.2
	Schistosomiasis (*n* = 8)	50	37.5	25	12.5	0	25	25	0	12.5	12.5	0	25
	Hepatitis A/E (*n* = 1)	0	100	0	0	0	0	0	0	100	0	0	0
	Soil-transmitted helminths (*n* = 13)	30.8	76.9	7.7	0	7.7	15.4	38.5	0	23.1	7.7	30.8	7.7
**Sexually-transmitted infections**	HIV/AIDS (*n* = 35)	40	82.9	22.9	8.6	17.1	0	25.7	22.9	28.6	2.9	42.9	20
	Syphilis (*n* = 7)	42.9	71.4	14.3	14.3	14.3	14.3	14.3	0	57.1	0	14.3	14.3
	Unplanned pregnancies (*n* = 12)	41.7	66.7	8.3	33.3	16.7	0	50	8.3	16.7	0	0	8.3
	Gonorrhea (*n* = 8)	25	87.5	12.5	0	12.5	0	37.5	0	50	0	12.5	12.5
**Zoonoses**	Any zoonotic disease (*n* = 6)	16.7	33.3	16.7	0	0	0	16.7	0	16.7	16.7	16.7	83.3
**Non-communicable diseases**	Cardio-vascular diseases (*n* = 14)	35.7	71.4	14.3	14.3	0	14.3	28.6	0	21.4	7.1	28.6	14.3
	Cancer (*n* = 7)	14.3	85.7	14.3	0	0	0	28.6	0	14.3	0	57.1	14.3
	Diabetes (*n* = 11)	27.3	81.8	27.3	9.1	0	0	45.5	0	18.2	9.1	18.2	18.2
	Chronic respiratory diseases (*n* = 13)	15.4	84.6	15.4	0	0	0	30.8	0	38.5	0	23.1	15.4
**Accidents and injuries**	Traffic-related injuries (*n* = 18)	27.8	77.8	16.7	16.7	5.6	0	27.8	0	27.8	0	38.9	11.1
	Work-related injuries (*n* = 7)	28.6	85.7	0	0	28.6	0	42.9	0	42.9	0	28.6	0
	Interpersonal violence (*n* = 10)	40	50	30	20	10	0	20	0	10	0	20	20
**Food- and nutrition-related issues**	Anemia (*n* = 14)	35.7	71.4	14.3	0	14.3	14.3	42.9	7.1	28.6	0	7.1	28.6
	Undernutrition (*n* = 21)	23.8	71.4	23.8	14.3	4.8	14.3	33.3	14.3	23.8	0	23.8	23.8
	Overweight (*n* = 4)	50	75	25	25	25	25	25	25	25	25	25	0
	Food-borne diseases (*n* = 4)	25	100	25	0	0	0	75	0	50	0	0	0
**MNCH**	Child immunization (*n* = 17)	29.4	82.4	11.8	5.9	11.8	0	23.5	17.6	29.4	5.9	29.4	17.6
	Maternal mortality (*n* = 15)	13.3	93.3	6.7	6.7	6.7	0	33.3	13.3	46.7	0	20	13.3
	Child mortality (*n* = 18)	5.6	94.4	0	0	5.6	0	27.8	16.7	44.4	0	27.8	16.7
**Mental health**	Anxiety/depression (*n* = 4)	50	75	25	25	25	0	0	0	25	0	50	0
	Self-harm/suicide (*n* = 0)	n.a.	n.a.	n.a.	n.a.	n.a.	n.a.	n.a.	n.a.	n.a.	n.a.	n.a.	n.a.

CDs = communicable diseases; Env. = environmental; MCH = maternal and child health; MNCH = maternal, neonatal and child health; n.a = not applicable; trad. = traditional. Percentages are illustrated on a color scale from red to blue. Red shading indicates percentages below 50%, blue shadings above 50%.
