# Supplementary material for: Inclusion of Health in Impact Assessment: A Review of Current Practice in Sub-Saharan Africa
Source: Int J Environ Res Public Health. 2020 Jun 10;17(11):4155. doi: 10.3390/ijerph17114155 (PMC7312242; doi:10.3390/ijerph17114155)
Supplement: Supplementary file 1 [file ijerph-17-04155-s001.zip › Supplementary file S1.pdf]

Dear Sir or Madam,

I am a PhD candidate in Epidemiology at the Swiss Tropical and Public Health Institute in Basel, Switzerland. Together with partners in Africa and Switzerland, we are conducting a research project aiming at understanding the impacts of resource extraction projects on health-related aspects of the Sustainable Development Goals in sub-Saharan Africa (https://www.swisstph.ch/fr/projects/hia4sd-health-impact-assessment/). My current research focus with in the project is the study of impact assessment reports with the aim to understand how different health aspects (e.g. health determinants, collaboration with health systems, diseases of concern) are included in the current impact assessment practice of the resource extraction sector.

Therefore, I am kindly asking you whether you are willing to share the impact assessment reports (e.g. environmental impact report, health impact report, etc.) that have been drafted as part of the feasibility studies of the PROJECTCOUNTRY with me. The reports will solely be used for research purposes and will not be shared with people outside our research group. We will not disclose any information about individual companies or project, nor disclose the location thereof. Instead, we will present summarised findings on the content level that do not allow any inferences about specific companies or projects. If requested, we are willing to sign a non-disclosure agreement.

I would be very grateful for your reply on my inquiry to clarify any remaining questions and the next steps for sharing the documents.

Many thanks for considering my request.

Kind regards,
