# Supplementary material for: Inclusion of Health in Impact Assessment: A Review of Current Practice in Sub-Saharan Africa
Source: Int J Environ Res Public Health. 2020 Jun 10;17(11):4155. doi: 10.3390/ijerph17114155 (PMC7312242; doi:10.3390/ijerph17114155)
Supplement: Supplementary file 1 [file ijerph-17-04155-s001.zip › Supplementary file S2.pdf]

Dear Sir or Madam,

I am a PhD candidate in Epidemiology at the Swiss Tropical and Public Health Institute in Basel, Switzerland. My current research is focusing on health in the context of natural resource extraction projects in sub-Saharan Africa. As a part of the research, I am screening impact assessment reports with the aim to understand how different health aspects (e.g. health determinants, collaboration with health systems, diseases of concern) are included in the current impact assessment practice of the natural resource extraction sector.

Therefore, I am kindly asking you whether you are willing to share the impact assessment reports (e.g. environmental impact report, health impact report, etc.) of resource extraction projects realised in COUNTRY. The reports will solely be used for research purposes and will not be shared with people outside our research group. We will not disclose any information about individual companies or project, nor disclose the exact location thereof. Instead, we will present summarised findings on the content level that do not allow any inferences about specific companies or projects.

In the end, the results will also be shared with you to inform you how health impacts are currently being assessed in the resource extraction sector in COUNTRY.

I would be very grateful for your reply on my inquiry to clarify any remaining questions and the next steps for sharing the documents.

Many thanks for considering my request.

Kind regards,
